# Polygenic disease risk scoring and genetic non-discrimination

**DOI:** 10.1093/jlb/lsaf016

**Published:** 2026-05-14

**Authors:** Jin K Park, Susannah Baruch, I Glenn Cohen

**Affiliations:** Yale Law School, 127 Wall Street, New Haven, CT 06511, United States; Petrie-Flom Center for Health Law Policy, Biotechnology, and Bioethics at Harvard Law School, 23 Everett St, Cambridge, MA 02138, United States; Petrie-Flom Center for Health Law Policy, Biotechnology, and Bioethics at Harvard Law School, 23 Everett St, Cambridge, MA 02138, United States; Harvard Law School, 1525 Massachusetts Ave, Cambridge MA 02138, United States

**Keywords:** genetic discrimination, polygenic risk scores, GINA, ADA

## Abstract

The Genetic Information Nondiscrimination Act (GINA) became law almost two decades ago, when genomic medicine was still in its infancy. One reason for its passage was to ensure that individuals and society would reap the benefits of emerging advances in genetic medicine, and would be able to benefit from genetic testing and research without fear of employment or health insurance discrimination. Since then, genomics has matured into a complex probabilistic science that increasingly allows for individualized estimates of genetic risk derived from large-scale population studies. Polygenic risk scores (PGSs), which provide genome-wide estimates of disease liability and may help indicate effective preventive care for an individual, raise new benefits but also concerns. PGS testing may become common in clinical practice, particularly to mitigate common complex diseases such as cardiac conditions and cancer. But are existing antidiscrimination protections adequate for a world where polygenic risk scoring is the norm? In this paper, we consider how existing laws apply and whether new legal and policy approaches are needed to support widespread, beneficial clinical use of PGSs. We also propose avenues for potential action by policymakers.

## I. INTRODUCTION

We are now witnessing a proliferation of scientific efforts that take full advantage of human genetic variation to elucidate the genetic architecture of complex diseases and phenotypes, the most prominent of which is the practice of polygenic disease risk scoring, which provides genome-wide estimates of disease liability. The development and clinical translation of polygenic risk scores (PGSs) is a fast-moving area of research with significant implications for the healthcare system. There is ongoing methodological innovation to improve the construction of PGSs driven in part by questions about their accuracy. While there is inherent complexity in predicting common diseases, investigators have been wary of asserting that PGSs are transferrable across populations given that the underlying data heavily draw from individuals of European ancestry.^1^ However, when polygenic scoring schemes are combined with rarer variants and highly penetrant monogenic mutations, they can provide increasingly robust estimates of genetic liability across ancestral groups.^2^ In addition, non-genetic health information may be added to the mix to help provide more accurate risk estimates. Indeed, population geneticists, physicians, and direct-to-consumer industry leaders have begun advocating for the sophisticated and responsible use of PGSs,^3^ particularly given improvements in their accuracy and demonstrations of clinical validity.^4^ One company developing a PGS for cardiovascular disease has reportedly already filed for Food and Drug (FDA) approval.^5^

One important question is whether PGS testing is covered by well-established legal frameworks that prohibit discrimination on the basis of genetics. For now, existing empirical studies suggest that US physicians and providers are particularly concerned with the exacerbation of health disparities traceable to the lack of portability of PGSs across ethnic ancestries, rather than outright employment discrimination.^6^ However, in 2021, the Polygenic Risk Score Task Force of the International Common Disease Alliance argued that the rapid adoption of PGSs ‘strengthens the case for stronger protections against genetic discrimination’, citing employment, educational, and insurance discrimination on the basis of sickle status in the USA during the 1970s.^7^

In the USA, the main statute that prohibits genetic discrimination is the Genetic Information Nondiscrimination Act (GINA), which prohibits discrimination based on an individual’s genetic information in both employment and health insurance. Congress passed GINA nearly unanimously, and it was signed into law by President George W. Bush in 2008. The statute’s goal was to address fears about genetic discrimination, allowing Americans to feel comfortable participating in research and to benefit from genetic medicine. As we discuss below, other anti-discrimination laws overlap or intersect with GINA’s protections, particularly the 1990 Americans with Disabilities Act (ADA) and the Americans with Disabilities Act Amendments Act of 2008 (ADAAA).

In this article, we consider how these existing federal antidiscrimination statutes, particularly GINA and the ADA as amended by the ADAAA, apply to discrimination on the basis of PRSs. Through three case studies, we identify and explore possible gaps in existing antidiscrimination statutes adopted before polygenic testing was explicitly contemplated. We focus on the novel questions posed by polygenic scoring in the context of employment, assessing protection from discrimination on the basis of an individual’s known genetic risk, what happens when the disease identified by the PGS may have ‘manifested’ in the mildest or earliest form, and whether an individual might seek accommodation at work in their quest to mitigate their genetic risk.

We show that widespread adoption and benefits of PGSs may depend upon the specifics of how courts apply these statutes. We aim to provide a nuanced road map for policymakers interested in the potential of this emergent area of medicine to improve human health. We hope to assist researchers, clinicians, legislators, policymakers, and others to advance the development, uptake, and utility of these cutting-edge tools in a responsible, ethical, and lawful manner.

## II. THE LEGAL LANDSCAPE RELEVANT TO PGS

*GINA* became law in 2008, prohibiting employers and health insurers from using genetic information to make decisions about hiring, firing, or coverage, based on the person’s genetic test results or family history. In its current form, GINA protects from discrimination only in the arenas of employment and health insurance. It does not protect against discrimination on the basis of diagnosed and/or manifested disease. ‘Manifested’ is defined as follows^8^:

Manifestation or manifested means, with respect to a disease, disorder, or pathological condition, that an individual has been or could reasonably be diagnosed with the disease, disorder, or pathological condition by a health care professional with appropriate training and expertise in the field of medicine involved. For purposes of this part, a disease, disorder, or pathological condition is not manifested if the diagnosis is based principally on genetic information.^9^

In essence, GINA prohibits discrimination based on genetic information, including family history, about conditions that have not yet manifested. Thus, GINA prohibits health insurers from denying coverage or adjusting premiums based on genetic information or requesting that an individual undergo a genetic test in the group or individual market, although an insurer may request that an individual undergo a test relevant to a coverage decision.^10^ Before GINA, state laws provided some protection from genetic discrimination, but they varied widely. GINA set a national ‘floor’ of protection but does not preempt state laws that provide more extensive protection.^11^

GINA was introduced in an era before PGSs and thus was not designed with PGSs in mind. The result is some difficult questions of fit, discussed below, but we also believe that a fair reading of the statute does answer some threshold questions about GINA’s relationship to PGSs. First, GINA does not prohibit *clinicians* from using PGSs to guide clinical management or for prospective screening, even if obtained by the individual consumer through Direct-to-Consumer testing. The statute makes clear that a healthcare professional providing care to a patient can request that the patient undergo genetic testing.^12^ Second, GINA does clearly prohibit an insurer or employer from using a PGS constructed using solely genetic information—test results or family history—in a way that is discriminatory, just as it would prohibit such use of the simpler forms of genetic testing that were available when the statute was first passed. Third, while GINA does not explicitly address this question, we believe that GINA also prohibits an employer or insurer’s use of PGSs, even in cases where some data points in the PGS calculation are *not* ‘genetic’ under GINA. Both the text and the stated purpose of the law support this reading, as we discuss later.

The *ADA* prohibits discrimination against individuals with disabilities in a wide range of contexts including employment. The ADA defines a disability as ‘a physical or mental impairment that substantially limits one or more major life activities; has a record of such an impairment; or is regarded as having such an impairment.’^13^ That third possibility, referred to as the ‘regarded as’ prong, is particularly relevant to genetic discrimination. In 1995, before the passage of GINA, the Equal Employment Opportunity Commission—the federal agency charged with enforcing various employment discrimination statutes— issued guidance advising that individuals discriminated against based on genetic information would be ‘regarded as’ having a disability.^14^

Responding to a series of Supreme Court opinions narrowly interpreting the ADA’s definition of disability, Congress passed the ADAAA^15^ seeking to change some elements of the way federal courts interpreted coverage for disability discrimination claims because plaintiffs in these cases had a significant loss rate in federal courts.^16^ Even if compelling evidence showed an employer had discriminated against the employee because of the impairment, courts would fail to vindicate their ADA claims if the condition in question did not fall into the narrow definition of disability. As one example, in *Murphy v United Parcel Service, Inc.*, the Supreme Court found that an individual with severe high blood pressure controlled by medication did not have a disability and thus could not pursue an ADA claim despite evidence he was fired because of his high blood pressure. The ‘Findings and Purposes’ section of the ADAAA explicitly made clear that the ADA’s definition of disability should be understood broadly.^17^ However, although employers must provide reasonable accommodations to qualified individuals with disabilities that meet the definition of disability in the statute, EEOC has clarified that ‘[u]nder the ADAAA, an employer *need not* provide a reasonable accommodation to an individual who meets only the “regarded as” prong of the definition of “disability”.’

In the following sections, we ask how this complex web of statutes applies to PGSs, focusing on the employment context. Through the use of three hypothetical cases we explore how discrimination and accommodation laws in employment play out for an individual who has a genetic risk discovered through a PGS and seeks to minimize their risk on the job. In this paper, we focus on GINA and the ADA as amended by the ADAAA, analyzing each hypothetical case under each law. However, it is worth noting that before GINA became law, the Health Insurance Portability and Accountability Act (HIPAA) of 1996^18^ provided some limited restrictions on the use of genetic information in premium adjustments and eligibility determinations for individuals in the group health insurance market (ie those receiving health insurance through an employer). HIPAA did not prevent health insurers from charging the employer more for group insurance, nor did it provide coverage in the individual health insurance market.

## III. PGS AND WORKPLACE PROTECTIONS

For employees who learn from a PGS that they have a particular risk of disease, how to navigate that information in the workplace is not obvious.

Case 1:

‘Alicia’ is a 30-year-old female working at a factory that manufactures chemicals. She is asymptomatic and is otherwise healthy. At the suggestion of her physician, she undergoes a genetic test and receives a polygenic risk score that puts her at significantly higher genetic risk of idiopathic pulmonary fibrosis (IPF) compared to other individuals of her age cohort. Her employer at the factory discovers the results of her test, and fires her on the basis of the test results.

In this case, Alicia likely has antidiscrimination protections under GINA. As the main federal statute governing discrimination on the basis of genetic information, GINA defines genetic information as ‘information about—(i) such individual’s genetic tests, (ii) the genetic tests of family members of such individual, and (iii) the manifestation of a disease or disorder in family members of such individual.’^19^ Title I of GINA prohibits the use of genetic information by health insurers in underwriting and Title II forbids the use of genetic information in employment, in hiring, firing, or promotion decisions. Both titles also include prohibitions against the acquisition of genetic information. As we argue in depth later in this paper, although GINA excludes certain features of individuals such as sex, age, and certain test results of proteins and other metabolites, the mere inclusion of this or other minor clinical information in calculating a polygenic risk score would not and should not prevent GINA from applying.^20^

As noted above, the text of the statute (as well as litigated cases and the EEOC’s interpretation of GINA) excludes protection from discrimination on the basis of conditions that have already manifested in the individual, ‘requir[ing] that genetic information be predictive or communicate actual medical risk’.^21^ Alicia is asymptomatic, she has not yet manifested the symptoms of disease, preserving GINA’s protections. Therefore, Alicia could not be fired on the basis of her PGS.

Alicia could also bring a claim under the ADA. She has no current disability as defined by the ADA, so the ADA’s ‘regarded as’ clause would apply if Alicia could prove that the firing was because she was regarded as disabled by the employer because of her PGS, in the absence of symptoms or manifestation.

Case 2:

‘Barry’ is a 30-year-old male working as a construction worker. At the suggestion of his physician, and as part of an annual physical examination, he undergoes a polygenic screening test and receives a PGS that puts him at significantly higher genetic risk of coronary heart disease (CHD) compared to other individuals of his age cohort. Although he is asymptomatic, reducing his heightened risk of CHD requires that he limit his exposure to job stressors, especially tasks with high effort-reward imbalance.^22^ As such, he hopes to receive workplace accommodations along these lines. Barry’s employer does not fire him, but refuses to provide accommodations.

Do existing anti-discrimination laws like GINA and the ADA help a worker pursue a reasonable workplace accommodation that may be necessary to reduce their risk once they learn of it? Let us consider both GINA and the ADA in turn.

Nothing in the text or legislative history of GINA suggests it was intended to provide mechanisms for employees to compel reasonable accommodations from an employer, even for reasons related to genetic testing. GINA will not be helpful in this regard.

For the ADA to apply, the question is whether Barry has a disability, a functional impairment that has impacted a major life activity, or alternatively, whether the ADA applies because he is ‘regarded as’ disabled. Because Barry is asymptomatic, his current symptoms likely do not rise to the level of a disability disrupting a major life activity most commonly claimed under the ADA. Barry might turn to the ADA’s ‘regarded as’ prong, which could protect Barry from being fired on the basis that the employer regards him as disabled, even though the employer is incorrect. However, even if Barry can prove the employer in fact has regarded him as a person with a disability, being ‘regarded as’ disabled does *not* give Barry the right to an accommodation such as a change in his working conditions or assignments. As discussed above, no accommodation is required under the ADA’s ‘regarded as’ clause.

It is clear from both Alicia’s and Barry’s cases that the PGS may cause the employer to regard an employee as disabled. Thus, an important implication of our analysis is that polygenic scoring may expand the class of individuals ‘regarded as’ having future impairments on the basis of genetic factors, but who cannot claim rights to accommodation that would reduce their risk as to that future impairment. The individual may be able to bring an ADA claim against an employer who refuses to hire the worker, fire the worker, or change the conditions of employment, but not successfully sue for an accommodation. Workers may voluntarily provide this information and collaborate with their employers to create such an accommodation, but they cannot rely on the ADA (or GINA) to *compel* their employer to provide reasonable accommodations. Neither GINA nor the ADA seems to provide a path for workers seeking accommodations to mitigate their prospective health risks.

Case 3a and Case 3b:

‘Charlie’ is a 30-year-old male working at a factory that manufactures chemicals. He has recently been having some mild dyspnea that intermittently influences his daily functioning, but is otherwise healthy. At the suggestion of his physician, he undergoes genetic testing and receives a PGS that puts him at significantly higher genetic risk of IPF compared to other individuals of his age cohort. Reducing his already heightened risk of IPF requires that he reduce his exposure to chemical irritants. As such, he hopes to receive workplace accommodations and voluntarily reveals his PGS to his employer to justify his request.

In Case 3a, Charlie’s employer receives Charlie’s request and does not fire him but refuses to provide accommodations.

In Case 3b, Charlie’s employer receives Charlie’s request and soon fires him, citing poor job performance.

Charlie has symptoms that seem related to the disease in question, but with a minimal impact on his daily functioning. It is quite possible a court would decide that the disease in question, IPF, has manifested even if the symptoms are mild and do not create a functional impairment*.*

GINA prohibits discrimination on the basis of genetic information rather than on the basis of symptoms and GINA does not require accommodations, so in Case 3a GINA would not provide relief. In Case 3b, GINA’s employment protections could apply if a court found Charlie was fired not on the basis of the poor job performance (whether or not impeded by his mild symptoms) but on the basis of the future risk revealed by the PGS. Similar unusual cases occur with traditional genetic testing: an employee is diagnosed with breast cancer and learns from genetic testing that they have a mutation that puts them at very high risk of other more deadly cancers and is fired. GINA prohibits discrimination on the basis of the genetic test result.

The ADA may not help Charlie either. The mild symptoms, even if related to IPF, do not seem to impair a major life activity. Therefore, Charlie likely would not be ‘disabled’ under the ADA and would not succeed in a lawsuit aimed at receiving accommodations (3a) or successfully challenge his firing (3b). That said, if Charlie can prove that he was ‘regarded as’ disabled by the employer, the ADA could protect Charlie from being fired (3a) if he can prove his job performance was acceptable, but his employer would not be required to provide accommodations to mitigate the risk revealed by the PGS (3b) because, as discussed above, lawsuits under the ‘regarded as’ prong cannot force an employer to provide accommodations.

Importantly, if the employer argues that it took action in Charlie’s case because the PGS suggested Charlie is more likely to develop a more serious disability in the future, Charlie may not be able to succeed in his lawsuit under the ADA. First, under Supreme Court precedent,^23^ if a PGS reveals that continued employment in a role would make an employee a direct threat to himself or others, that may also provide a defense to the employer under the ADA for an adverse employment action. Second, although the Supreme Court has not ruled on this question, several circuit courts have found that the ADA does not apply when an employer discriminates on the belief that a future condition may develop.^24^ In two recent cases, the US Court of Appeals for the Seventh Circuit ruled that obesity alone is not a disability in itself absent an underlying physiological cause, and that the ADA does not protect from employer discrimination on the belief that obesity increases the risk of future impairment, even if that future impairment could meet the statute’s requirement of a disability.^25^ In addition, in *Darby v Childvine, Inc.,* the US Court of Appeals for the Sixth Circuit held that for a condition to qualify as a disability under ADA, a major life activity must have been disrupted, stating: ‘A genetic mutation that merely predisposes an individual to other conditions, such as cancer, is not itself a disability under the ADA.’^26^ That decision surprised some observers who have assumed, especially since the passage of the ADAAA, that the ‘regarded as’ prong includes those who may have an impediment in the future.^27^

### III.A. Is Genetic Predisposition Alone a Disability under the ADA?

The Supreme Court has never ruled on whether a genetic predisposition alone is a disability under the ADA.^28^ In *Bragdon v Abbott* the Court ruled that the plaintiff—an asymptomatic HIV-positive patient denied dental services in a dentist’s office who was asked to bear the additional cost of having her cavity filled in a hospital—was covered by the ADA. The plaintiff argued being HIV-positive was a substantial limitation on her reproduction because she could potentially transmit the virus to a partner or child. The Court agreed the risk was a substantial limitation on reproductive capabilities, even if it was possible for her to conceive, and agreed that reproduction qualified as a major life activity under the ADA.^29^ In *Bragdon*, the Court ruled that individuals with HIV infection may be covered under the ADA even if they are free of symptoms or have no AIDS diagnosis because their reproduction is nevertheless affected. The Court did not address whether someone impacted by a progressive condition who does not *currently* face a substantial barrier to reproduction or another major life activity is covered by the ADA. In a dissenting opinion written by then-Chief Justice Rehnquist, ‘Taken to its logical extreme, [the argument] would render every individual with a genetic marker for some debilitating disease “disabled” here and now because of some possible future effects.’

Increased use of PGSs could put this question front and center. In some cases, the PGS might indicate that the individual is at increased risk related to pregnancy and—consistent with *Bragdon*—that the condition affects the major life activity of reproduction, rendering the individual disabled within the meaning of the statute. Or the results of the PGS may indicate that the individual's offspring would be at higher risk for the disease, again limiting the major life activity of reproduction. Both arguments are, as far as we know, untested in the courts. Such arguments would raise additional difficult questions, including how much of a risk to the individual or offspring would be needed to make the person receiving the PGS result ‘disabled’ within the meaning of the statute.

### III.B. Composite Scores: Why GINA Applies

For GINA specifically, there is a threshold question of what kinds of PGSs are a ‘genetic test’ within the meaning of the statute. While the most common PGS on the market is a score generated from data obtained by SNP genotyping arrays, after which an individualized score is constructed from an algorithm trained on a separate training dataset,^30^ it is likely that these scores will include various non-genetic risk factors (age, diet, smoking status, BMI, laboratory values, family history, exercise, etc.) as they enter clinical testing and eventual integration into electronic health systems. Sophisticated disease risk scoring schemes seek to incorporate various non-genetic risk factors such as clinical and laboratory data from health records, environmental risk factors, and lifestyle risks to create composite risk scores. Several studies have demonstrated that incorporating well-established clinical risk factors into PGSs may significantly improve clinical risk prediction.^31^ PGSs have even been added to improve the predictive value of pooled cohort equations, which are commonly used to guide preventive measures and clinical practice guidelines.^32^ There are potentially transformative benefits that may attach to incorporating non-genetic factors, given that such a combination could identify individuals with particularly high genetic and environmental disease risk who could then receive targeted screening. Combining both the genetic and non-genetic risk factors to create a composite polygenic score represents the cutting edge of the current science around polygenic scoring.

In our view, use of a combined or composite score to discriminate in employment or health insurance clearly violates antidiscrimination protections under GINA. The definition of what is protected by GINA is broad. For example, it includes not only the individual’s genetic test results, but also their family history up to and including the genetic test results of up to a fourth-degree relative. It also includes the manifestation of disease in the tested party's relative, or the genetic tests of any fetus of an individual or family member. It also includes ‘any request for, or receipt of, genetic services, or participation in clinical research which includes genetic services, by such individual or any family member of such individual.’

The drafters of GINA understood that to effectively protect individuals from genetic discrimination, they had to include a broad swath of information. Today, PGSs predict complex diseases based on many genetic data points and they are most predictive when they include some clinical data, particularly for conditions with low genetic heritability. Some data included in PGSs may not be included in the law’s definition of genetic information. Some may even be explicitly excluded, such as sex, age, and certain test results of proteins and other metabolites. But we believe that at its core, a PGS is a result predicting complex diseases based on genetic information; thus, discrimination on the basis of the score itself would be prohibited by GINA.^33^ Despite the multifactorial nature of composite scores, genetic information is still genetic information, and its protections do not somehow become blunted with the addition of *othe*r types of information. Any score that includes genetic information in the manner defined by the statute will trigger its protections. As Congress stated in its findings introducing GINA, ‘Federal legislation...is necessary to fully protect the public from discrimination and allay their concerns about the potential for discrimination, thereby allowing individuals to take advantage of genetic testing, technologies, research, and new therapies.’^34^ Our reading of the statute is consistent with this purpose.

PGSs from one of the proliferating direct-to-consumer companies currently offering tests for a variety of conditions should also be considered protected by GINA, whether they include some non-genetic information or not. These scores may become known to the individual’s health insurer or employer if they seek preventive care or accommodation at work. Whether voluntarily shared or not, these scores include genetic information as part of disease risk assessment, and any use by an employer or health insurer to make employment decisions or adjust premium prices would violate antidiscrimination protections under GINA.^35^

As we described above, some types of information that are not purely ‘genetic’ in nature—eg family history—are explicitly included in GINA's definition of genetic information. At the same time, other types of information—specifically sex—have been carved out of the definition of genetic information in the statute.^36^ Because of this, the extent to which these non-genetic disease risk scoring schemes run afoul of GINA will depend on what kind of information is being included, and how much of it might be deemed non-genetic.

### III.C. Legislative and Policy Responses

Major developments in the field of genomic medicine are likely to put pressure on the existing statutory scheme for genetic nondiscrimination. To the extent legislators or other policymakers may be open to considering updating the existing statutory schemes, some changes would be helpful to consider.

First, as we have discussed, we believe polygenic scoring is and should be covered by GINA on any plausible reading of that statute’s requirements. The core component of a PGS is *genetic* and GINA prohibits employers or insurers from utilizing that information as the basis of decision-making. However, it may be useful for legislators to clarify that GINA covers risk-scoring schemes even if non-genetic information is included, so long as the PGS as a whole is purely predictive (ie a scenario where the individual has no symptoms). This would harmonize predictive information across genetic and non-genetic contexts, which many commentators have advocated for in the years since GINA’s passage.^37^ Questions about entirely non-genetic disease risk scores would also need to be resolved.

Second, as demonstrated in our cases above, an asymptomatic employee who seeks work accommodations to prevent or mitigate early sequelae of workplace exposure for a known disease risk based on a polygenic test has little recourse in the ADA or GINA. Policymakers could consider how law and regulations might help individuals benefit from the information gained through PGS by accessing work conditions that do not exacerbate their particular risks.

An optimistic view of PGSs suggests that these scores have the potential to improve individual and public health. That outcome will require widespread uptake and depend on already-stretched clinicians explaining to patients the relationship between PGS and disease. Professional organizations that govern the standards of medical practice will need to define how these scores are used and interpreted. In our current context of widespread skepticism of science and public health expertise, we hope for responsible, deliberate, and participatory consideration of how to ensure broad benefits from innovation in genetic medicine and disease prevention.

## Footnotes

1 A. C. F. Lewis & R. C. Green, *Polygenic Risk Scores in the Clinic: New Perspectives Needed on Familiar Ethical Issues*, 13 Genome  Med.14 (2021), https://doi.org/10.1186/s13073-021-00829-7; A. R. Martin, M. Kanai, Y. Kamatani et al., *Clinical Use of Current Polygenic Risk Scores May Exacerbate Health Disparities*, 51 Nat  Genet. 584–91 (2019), https://doi.org/10.1038/s41588-019-0379-x; A. C. Palk, S. Dalvie, J. de Vries et al., *Potential Use of Clinical Polygenic Risk Scores in Psychiatry – Ethical Implications and Communicating High Polygenic Risk*, 14 Philos. Ethics  Humanit. Med. 4 (2019), https://doi.org/10.1186/s13010-019-0073-8; Y. Wang, K. Tsuo, M. Kanai et al., *Challenges and Opportunities for Developing More Generalizable Polygenic Risk Scores*, 2022 Annu. Rev. Biomedic. Data  Sci. 30 (2022).

2 A. P. Patel, M. Wang, Y. Ruan et al., *A Multi-ancestry Polygenic Risk Score Improves Risk Prediction for Coronary Artery Disease*. 29 Nat. Med. 1793–803 (2023), https://doi.org/10.1038/s41591-023-02429-x; M. E. Weale, F. Riveros-Mckay, S. Selzam et al. *Validation of an Integrated Risk Tool, Including Polygenic Risk Score, for Atherosclerotic Cardiovascular Disease in Multiple Ethnicities and Ancestries*, 148 Am J Cardiol. 157–64 (2021), https://doi.org/10.1016/j.amjcard.2021.02.032; N. Kurniansyah, M. O. Goodman, A. T. Khan et al., *Evaluating the Use of Blood Pressure Polygenic Risk Scores across Race/Ethnic Background Groups*, 14 Nat. Commun. 3202 (2023), https://doi.org/10.1038/s41467-023-38,990-9; N. J. Lennon, L. C. Kottyan, C. Kachulis et al., *Selection, Optimization and Validation of Ten Chronic Disease Polygenic Risk Scores for Clinical Implementation in Diverse US Populations*, 30 Nat  Med. 480–87, https://doi.org/10.1038/s41591-024-02796-z.

3 H. Wand, S. A. Lambert, C. Tamburro et al., *Improving Reporting Standards for Polygenic Scores in Risk Prediction Studies*, 591 Nature 211–19 (2021), https://doi.org/10.1038/s41586-021-03243-6.

4 Patel et al., *A Multi-ancestry Polygenic Risk Score Improves Risk Prediction for Coronary Artery Disease*, 29 Nat  Med. 1793–803 (2023), https://doi.org/10.1038/s41591-023-02429-x; M. E. Weale, F. Riveros-Mckay, S. Selzam et al., *Validation of an Integrated Risk Tool, Including Polygenic Risk Score, for Atherosclerotic Cardiovascular Disease in Multiple Ethnicities and Ancestries*, 148 Am. J. Cardiol. 157–64 (2021), https://doi.org/10.1016/j.amjcard.2021.02.032; P. Pal Choudhury, M. N. Brook, A. N. Hurson et al., *Comparative Validation of the BOADICEA and Tyrer-Cuzick Breast Cancer Risk Models Incorporating Classical Risk Factors and Polygenic Risk in a Population-Based Prospective Cohort of Women of European Ancestry*, 23 Breast  Cancer  Res. 22 (2021), https://doi.org/10.1186/s13058-021-01399-7; M. Tamlander, N. Mars, M. Pirinen et al., *Integration of Questionnaire-Based Risk Factors Improves Polygenic Risk Scores for Human Coronary Heart Disease and Type 2 Diabetes*, 5 Commun  Biol. 1–13 (2022), https://doi.org/10.1038/s42003-021-02996-0.

5
*Revolutionary Study Offers Hope in Combating Coronary Heart Disease. | Genetic Testing For Personalised Health Care and Medicine*, Apr. 4, 2024, https://genincode.com/cardio-incode-score-clinical-publication-on-polygenic-risk-of-coronary-heart-disease/ (accessed Oct. 26, 2024).

6 B. J. Kerman, C. A. Brunette, E. J. Harris et al., *Primary Care Physician Use of Patient Race and Polygenic Risk Scores in Medical Decision-Making*, 25 Genet  Med. 100800 (2023), https://doi.org/10.1016/j.gim.2023.100800; A. C. F. Lewis, E. F. Perez, A. E. R. Prince et al., *Patient and Provider Perspectives on Polygenic Risk Scores: Implications for Clinical Reporting and Utilization*, 14 Genome  Med. 114 (2022), https://doi.org/10.1186/s13073-022-01117-8.

7 A. Adeyemo, M. K. Balaconis, D. R. Darnes DR, et al., *Responsible Use of Polygenic Risk Scores in the Clinic: Potential Benefits, Risks and Gaps*, 27 Nat  Med. 1876–84 (2021), https://doi.org/10.1038/s41591-021-01549-6.

8 J Roberts, I. Cohen, C. Deubert C & H. Lynch, *Evaluating NFL Player Health and Performance: Legal and Ethical Issues*, 165 U. PA. L. Rev. 227, at 286–287 (2017).

9 29 C.F.R. § 1635.3(g).

10 Genetic Information Nondiscrimination Act, Vol. 122 Stat. 881; 2008.

11 K. L. Hudson, M. K. Holohan & F. S. Collins, *Keeping Pace with the Times—The Genetic Information Nondiscrimination Act of 2008*, 358 New  Engl. J. Med. 2661–63 (2008), https://doi.org/10.1056/NEJMp0803964.

12 29 U.S.C. 1182(c)(2).

13 42 U.S.C. § 12102(1)(A).

14 Equal Employment Opportunity Commission, Compliance  Manual, Vol. 2, Section 902, Order 915.002,902-45 (1995).

15 ADA Amendments Act of 2008, https://www.eeoc.gov/statutes/ada-amendments-act-2008 (accessed Feb. 16, 2025).

16 E. Kaiser, *The Americans with Disabilities Act: An Unfulfilled Promise for Employment Discrimination Plaintiffs*, 2004 U. PA. J. Bus. L. 6.

17 Equal Employment Opportunity Commission, *ADA Amendments Act of 2008*, https://www.eeoc.gov/statutes/ada-amendments-act-2008 (accessed Feb. 16, 2025) (section 2).

18 Health Insurance Portability and Accountability Act, 1996.

19 42 U.S.C. § 2000ff(4)(A).

20 J. Roberts, *The Genetic Information Nondiscrimination Act as an Antidiscrimination Law*, 86 Notre  Dame L. Rev. 597 (2011).

21 B. A. Areheart & J. L. Roberts, *GINA, Big Data, and the Future of Employee Privacy*, 128 Yale L. J. 544–871, at 768 (2019).

22 M. Lavigne-Robichaud, X. Trudel, D. Talbot et al., *Psychosocial Stressors at Work and Coronary Heart Disease Risk in Men and Women: 18-Year Prospective Cohort Study of Combined Exposures*, 16 Circ. Cardiovasc. Qual. Outcomes e009700 (2023), https://doi.org/10.1161/CIRCOUTCOMES.122.009700.

23 Chevron U.S.A. Inc. v. Echazabal, 536 U.S. 73 (2002)

24 M. A. Rothstein, *GINA, the ADA, and Genetic Discrimination in Employment*, 36 J. L. Med. & Ethics 837 (2008), https://doi.org/10.1111/j.1748-720X.2008.00341.x.

25 Shell v. Burlington N. Santa Fe Ry. Co., 941 F.3d 331 (7th Cir. 2019); Richardson v. Chicago Transit Auth., 926 F.3d 881 (7th Cir. 2019).

26 Darby v. Childvine, Inc., 964 F.3d 440, 446 (6th Cir. 2020).

27 M. A. Rothstein, *Predictive Health Information and Employment Discrimination under the ADA and GINA*, 48 J. L. Med. & Ethics 595–602 (2020), https://doi.org/10.1177/1073110520958887.

28
*Id.* at page 598.

29 M. T. Friedland, *Not Disabled Enough: The ADA’s ‘Major Life Activity’ Definition of Disability*, 52 Stanford L. Rev. 171 (1999), https://doi.org/10.2307/1229460.

30 S. W. Choi, T. S. H. Mak & P. F. O’Reilly, *Tutorial: a Guide to Performing Polygenic Risk Score Analyses*, 15 Nat. Protoc. 2759–72 (2020), https://doi.org/10.1038/s41596-020-0353-1.

31 A. P. Patel, M. Wang M, Y. Ruan et al., *A Multi-ancestry Polygenic Risk Score Improves Risk Prediction for Coronary Artery Disease*, 29 Nat. Med. 1793–803 (2023), https://doi.org/10.1038/s41591-023-02429-x; M. Tamlander, N. Mars, M. Pirinen et al., *Integration of Questionnaire-Based Risk Factors Improves Polygenic Risk Scores for Human Coronary Heart Disease and Type 2 Diabetes*, 5 Commun  Biol. 1–13 (2022), https://doi.org/10.1038/s42003-021-02996-0; A. Torkamani, N. E. Wineinger & E. J. Topol, *The Personal and Clinical Utility of Polygenic Risk Scores*, 19 Nat. Rev. Genet. 581–90 (2018), https://doi.org/10.1038/s41576-018-0018-x; A. M. Small, G. E. M. Melloni, F. K. Kamanu et al., *Novel Polygenic Risk Score and Established Clinical Risk Factors for Risk Estimation of Aortic Stenosis*, 9 JAMA Cardiol. 357–66 (2024), https://doi.org/10.1001/jamacardio.2024.0011; N. J. Samani, E. Beeston, C. Greengrass et al., *Polygenic Risk Score Adds to a Clinical Risk Score in the Prediction of Cardiovascular Disease in a Clinical Setting*, 34 Eur. Heart J. 3152–60 (2024), https://doi.org/10.1093/eurheartj/ehae342; S. van Dam, P. Folkertsma, J. Castela Forte et al., *The Necessity of Incorporating Non-genetic Risk Factors into Polygenic Risk Score Models*, 13 Sci. Rep. 1351 (2023), https://doi.org/10.1038/s41598-023-27,637-w.

32 A. P. Patel, M. Wang, Y. Ruan et al., *A Multi-ancestry Polygenic Risk Score Improves Risk Prediction for Coronary Artery Disease*, 29 Nat  Med. 1793–803 (2023), https://doi.org/10.1038/s41591-023-02429-x; J. W. Groenendyk, P. Greenland & S. S. Khan, *Incremental Value of Polygenic Risk Scores in Primary Prevention of Coronary Heart Disease: A Review*, 182 JAMA Intern. Med. 1082–88 (2022), https://doi.org/10.1001/jamainternmed.2022.3171.

33 Genetic Information Nondiscrimination Act, Vol. 122, Stat. 881; 2008.

34
*Id.* at page 883.

35 J. S. Sherkow, J. K. Park & C. Y. Lu, *Regulating Direct-to-Consumer Polygenic Risk Scores*, 330 JAMA 691–92 (2023), https://doi.org/10.1001/jama.2023.12262; A. Regalado, *23andMe Thinks Polygenic Risk Scores Are Ready for the Masses, but Experts Aren’t So Sure*, MIT Tech. Rev., Mar. 8, 2019, https://www.technologyreview.com/2019/03/08/136730/23andme-thinks-polygenic-risk-scores-are-ready-for-the-masses-but-experts-arent-so-sure/ (accessed Oct. 3, 2022).

36 42 U.S.C. § 2000ff(4)(C).

37 R. C. Green, D. Lautenbach, A. L. McGuire, *GINA, Genetic Discrimination, and Genomic Medicine*, 372 N. Engl. J. Med. 397–99 (2015), https://doi.org/10.1056/NEJMp1404776; M. A. Rothstein, *Genetic Exceptionalism and Legislative Pragmatism*, 35 J. L. Med. & Ethics. 59–65 (2007). https://doi.org/10.1111/j.1748-720X.2007.00154.x

